# Improving functioning in severe mental illness with cognitive remediation: study protocol for a pragmatic randomised multi-centre trial with adjunct sham-controlled tDCS (HEADDSET+)

**DOI:** 10.1007/s00406-026-02245-7

**Published:** 2026-04-24

**Authors:** Nienke Catharina Buist, Anika Poppe, Branislava Ćurčić-Blake, Gerdina Hendrika Maria Pijnenborg, Lisette van der Meer

**Affiliations:** 1 Department of Clinical and Developmental Neuropsychology, University of Groningen, Grote Kruisstraat 2/1, 9712 TS Groningen, The Netherlands; 2 Department of Rehabilitation, Lentis Psychiatric Institute, Lagerhout E35, 9741 KE Zuidlaren, The Netherlands; 3 Research School of Behavioural and Cognitive Neurosciences (BCN), University of Groningen, Groningen, The Netherlands; 4 Department of Psychiatry, Donders Institute for Brain, Cognition and Behaviour, Radboud University Medical Center, Nijmegen, The Netherlands; 5 Department of BSCS Neuroscience, University of Groningen, University Medical Center Groningen, Groningen, The Netherlands; 6 Department of Psychotic Disorders, GGZ Drenthe, Assen, The Netherlands; 7 Rob Giel Research Center, University Center Psychiatry, University Medical Center, University of Groningen, Groningen, The Netherlands

**Keywords:** Cognition, Cognitive remediation, Daily functioning, Recovery, Severe mental illness, Transcranial direct current stimulation

## Abstract

Individuals with severe mental illness (SMI) face daily challenges, often due to cognitive impairments. While cognitive remediation (CR) can improve both cognitive and functional outcomes, especially when combined with other rehabilitation interventions, its effectiveness in individuals with SMI requiring sheltered housing remains unclear. This trial primarily investigates whether CR improves daily and cognitive functioning compared to a waitlist period. As a second exploratory aim, we will examine whether adding transcranial direct current stimulation (tDCS) enhances the effectiveness of CR in a randomised sham-controlled comparison. Lastly, the trial examines participants’ subjective experiences of the CR intervention. We hypothesise improvements in goal attainment, daily functioning, and cognition. Using a non-concurrent multiple baseline design, 126 participants (aged 18–65) with SMI residing in sheltered housing facilities will receive 16–20 weeks of twice-weekly CR sessions lasting 30–45 min, combined with either active (*N* = 63) or sham tDCS (*N* = 63). Functional, cognitive, and clinical assessments will be conducted at baseline, post-waitlist, post-treatment, and 6-month follow-up. Additionally, participants’ meta-cognitive skills and subjective experiences will be evaluated post-treatment. In multilevel mixed models, we will evaluate the effect of CR (combining both CR groups) vs. waitlist, and compare CR + active tDCS vs. CR + sham tDCS to explore potential augmentation effects. If effective, integrating CR, with or without tDCS, into standard care may benefit recovery in individuals with SMI requiring long-term support. ClinicalTrials.gov: NCT06378463, registered on April 17, 2024.

**Supplementary Information:**

The online version contains supplementary material available at 10.1007/s00406-026-02245-7.

## Background

People with severe mental illness (SMI) suffer from mental health problems associated with chronic and persistent challenges that substantially affect functioning across key life domains, including social, occupational, and housing areas, and require long-term intensive support [2, 3]. Most individuals with SMI are diagnosed with psychotic disorders [2]. Other common conditions include bipolar disorder, major depressive disorder, and substance-related disorders. Approximately 1.3% of the Dutch population lives with SMI, with 43% requiring long-term psychiatric care for six years or longer in settings such as sheltered housing or clinical facilities [4]. Individuals with SMI frequently experience persistent symptoms [5], difficulties with medication adherence [6], physical health problems [7], and challenges in self-care [2, 8]. These challenges may extend to daily functioning, such as managing household tasks, using public transportation, or occupational capabilities [9, 10].

Many functional difficulties are highly prevalent [11] and associated with cognitive impairments [12–16], particularly in memory, attention, and executive functioning (e.g., planning and organising) [17–19]. The most common treatment for schizophrenia, antipsychotic drugs, has the potential to reduce positive symptoms but has little beneficial effect on cognitive problems [20]. This has stimulated interest in interventions targeting (the impact of) cognitive impairments directly [21–25]. Recent studies indicate that about 40% of the individuals with schizophrenia achieve full recovery across clinical, functional and personal domains [26–28]. Interventions, particularly targeting cognitive functioning, may further support independence [29], recovery [30], and quality of life [31].

Cognitive remediation (CR) refers to a class of evidence-based behavioural interventions designed to improve cognition and thereby enhance daily functioning [32]. Beyond these gains, CR may also yield broader therapeutic benefits, including improvements in motivation, metacognitive awareness, and self-esteem, as well as alleviation of negative symptoms [33–36]. Meta-analyses demonstrate that CR significantly improves psychosocial functioning in people with schizophrenia [22, 24, 25, 37, 38], particularly when integrated with broader rehabilitation efforts that support real-world generalisation [21, 22]. CR has also shown promise across other diagnostic groups, including mood disorders [39], eating disorders [40], borderline personality disorder [41], and attention deficit hyperactivity disorder [42]. Although some evidence supports its effectiveness among individuals in forms of sheltered or long-term clinical accommodations, the literature remains scarce [43].

Decades of research have identified four core components critical to CR’s effectiveness: trained therapist guidance, strategy learning, repetitive practice, and transfer to daily functioning [23, 25, 44, 45]. The “Computerised Interactive Remediation of Cognition and Thinking Skills” (CIRCuiTS™, King’s College London) is such an intervention that incorporates all these components [44, 46]. This program has demonstrated effectiveness in people with psychotic disorders, but has not yet been applied in people with SMI in sheltered housing. Studying CR in this population is particularly relevant, given their high support needs and additional learning challenges.

Cognitive improvements resulting from CR have been linked to structural changes in the brain essential for retaining newly acquired skills [47], including increased network activation [48], and white and grey matter alterations [49, 50]. The brain’s ability to change and adapt neural connections in response to experiences or learning is called neuroplasticity and plays a critical role in maintaining and improving cognitive functioning and adaptation to new situations [51].

Research suggests that methods such as transcranial direct current stimulation (tDCS) may improve neuroplasticity. TDCS is a non-invasive brain stimulation (NIBS) technique that uses low electrical currents, typically 1–2 mA, and can be applied relatively easily with minimal side effects [52]. By applying electrodes to specific scalp areas, tDCS can selectively modulate brain activity and neocortical excitability in underlying cortical regions [52–54]. It potentially improves cognitive functioning by altering neurotransmission [55, 56] in schizophrenia [57, 58]. Of particular interest is the frontoparietal brain network (FPN), an interconnected brain region important in executing cognitive tasks [59], which is often affected in schizophrenia [60]. Within this network, the left dorsolateral prefrontal cortex (DLPFC) is identified as a key region mediating executive functions and a potential target for tDCS [61].

It has been suggested that combining CR with other methods that improve neuroplasticity, including NIBS, medication, or physical exercise, could result in more significant and longer-lasting improvements and reduce the duration of training protocols [62]. By combining cognitive training with tDCS, improvements in attention and working memory in neuropsychiatric disorders are observed, although effects on global cognition and language remain small and statistically nonsignificant [63]. However, current evidence regarding the effectiveness of combined approaches to improve cognition, particularly CR with NIBS, remains limited. This is mainly due to heterogeneity and methodological weaknesses, such as small group trials and insufficient assessment of real-world generalisation [64]. Moreover, evidence on whether any additive effects of tDCS translate to improvements in everyday functioning remains limited [64]. In a recent meta-analysis, tDCS emerged as the most frequently used NIBS technique in combination with CR [64]. Compared to repetitive transcranial magnetic stimulation (rTMS), tDCS is more cost-effective, portable, better tolerated, and can be applied concurrently with CR. Although tDCS has limited spatial focality, it may modulate distributed prefrontal networks that are relevant for CR. Nevertheless, empirical evidence supporting CR with tDCS augmentation, particularly in inpatient or long-term care settings and for everyday functioning outcomes, remains preliminary and requires further investigation.

We previously demonstrated the feasibility and acceptability of this combined approach in individuals with SMI in a pilot study [65, 66]. In that pilot, preliminary outcome patterns suggested improvements in a global cognition composite after CR and at follow-up, whereas between-group differences (active vs. sham) were not detected. Accordingly, the tDCS augmentation question is prespecified as a secondary and exploratory objective. The rationale for testing this combination in sheltered housing is based on the hypothesis that neuromodulation may support CR, alongside the established feasibility and safety, and substantial unmet clinical need in this population. Building on this work, the current multi-centre clinical trial primarily investigates the effectiveness of a CR intervention in individuals with SMI residing in sheltered housing, while secondarily comparing CR with augmented tDCS.

The primary objective is to assess whether participation in the CR programme leads to improved goal attainment, daily functioning, and cognitive functioning, compared to a waitlist control period in which participants receive treatment as usual (TAU). The second objective is to explore whether combining CR with augmented active tDCS improves the effects of CR combined with sham tDCS, as measured by the same outcome measures. We hypothesise that participants receiving CR will show significantly greater improvements in goal attainment, daily functioning, and cognitive functioning compared to those in the waitlist condition. Furthermore, we expect that the combination of CR with active tDCS will demonstrate significantly greater improvements across the same domains compared to CR with sham tDCS.

## Method

### Trial design

**Fig. 1:**
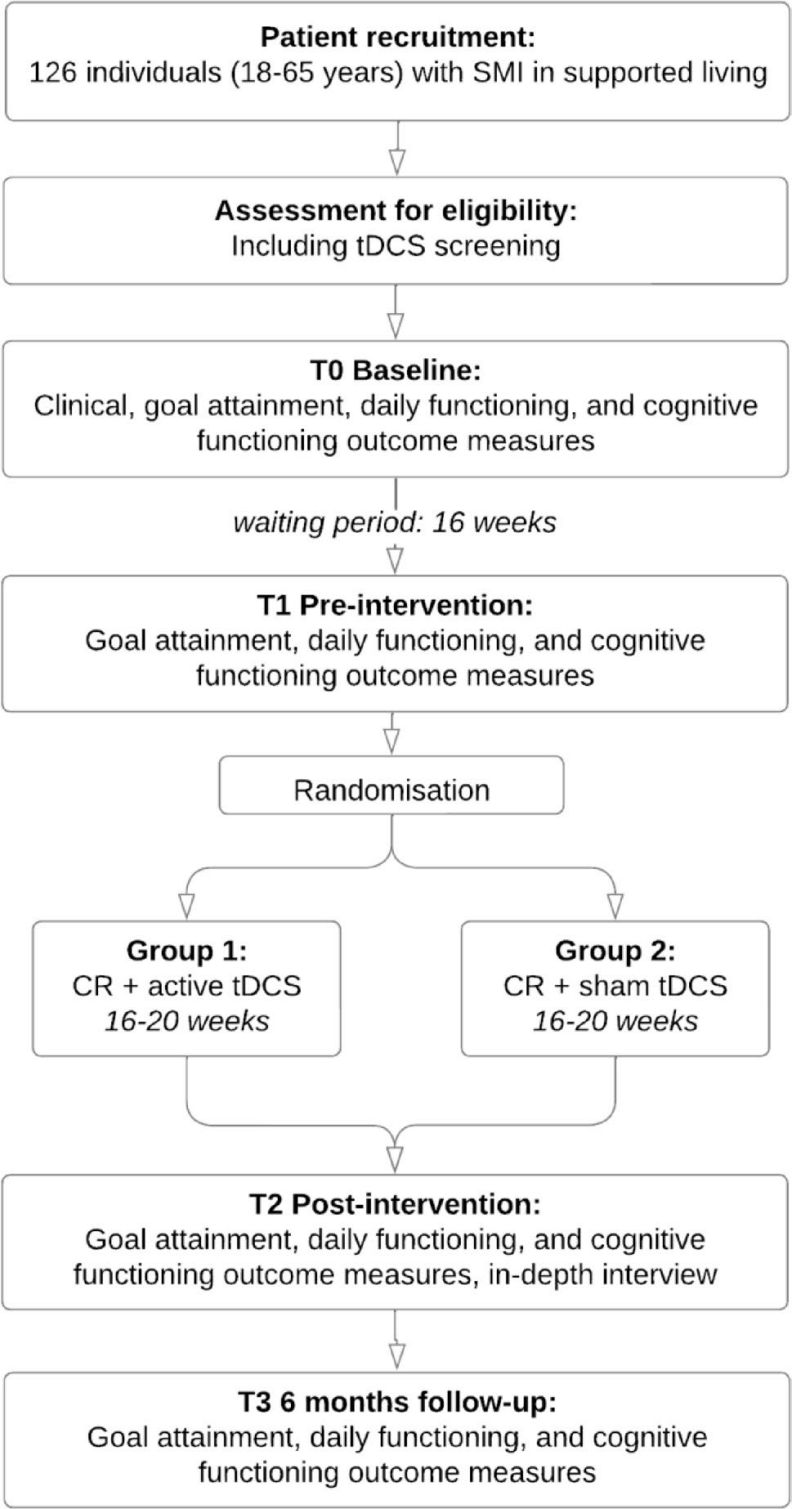
Flowchart of the study design

This pragmatic, triple-blinded, randomised, sham-controlled multi-centre trial follows a non-concurrent multiple baseline design (Fig. 1). Recruitment and eligibility assessment are followed by enrolment into five cohorts, each comprising approximately 20–24 participants, spaced 16–20 weeks apart. After inclusion, participants complete two baseline assessments (T0, T1), separated by a 16-week waiting period serving as a within-subject control, offering greater internal validity than a between-subject design. This design enables all participants to receive the intervention and attain its potential benefits. After T1, participants are randomised to either CR + active tDCS or CR + sham tDCS. After the 16-20-week intervention period, outcomes are reassessed post-treatment (T2, within 2 weeks) and at 6-month follow-up (T3).

### Study population

Eligible participants are individuals with SMI defined as follows: (1) having a psychiatric disorder requiring care or treatment (no remission of positive, negative, and cognitive symptoms); (2) severe disabilities in social and societal functioning (no functional remission); (3) disabilities resulting from a psychiatric disorder; (4) disabilities being structural (lasting at least several years); (5) coordinated professional care is necessary to realise a treatment plan [2, 3]. Participants must reside in sheltered housing facilities of a psychiatric institute, where staff provide continuous assistance (24/7) with daily activities when necessary. Additional inclusion criteria are: (1) age 18–65, (2) sufficient written and oral proficiency in Dutch, and (3) ability to provide informed consent. Exclusion criteria are: (1) having previously received CIRCuiTS, (2) contraindications for tDCS (e.g., intracranial metal or electronic implants, severe scalp skin lesions, history of seizures), and (3) substance use interfering with the participant’s typical level of functioning during training sessions.

Eligibility is determined through screening against predefined inclusion and exclusion criteria. Participants also complete a tDCS screening checklist. If any concerns arise (i.e., a ‘yes’ response to any of the questions), the researcher (NB) gathers additional information from the participant and, if needed, their clinician to determine whether it constitutes a contraindication for participation. Remaining uncertainties are discussed with the research team, including tDCS experts and clinicians.

### Study centres and recruitment

Participants are recruited from sheltered housing facilities at three mental health care organisations in the Northern Netherlands: Cosis, GGZ Friesland, and Lentis Psychiatric Institute. Recruitment involves collaboration with healthcare professionals at these institutes, who help identify potential participants and organise informative meetings. Eligible participants receive detailed oral and written information about the study and have at least one week to decide. Written informed consent is obtained before participation, and participants may direct any further questions to the researchers. Participants are informed about the randomised trial design, emphasising that group assignment is random and that improvement in goal attainment, daily functioning, or cognition cannot be guaranteed. Retention is promoted by maintaining regular contact with participants during the waitlist period, offering flexibility to accommodate participants’ preferences, and emphasising the potential benefits of the training. Withdrawal is possible at any time without consequences.

After completing the training, participants are invited to participate in an additional in-depth interview. They receive the same informed consent procedure and detailed information (i.e., time to consider participation, questions, consent, and withdrawal) only if they express interest. Researchers or CIRCuiTS therapists invite participants during or after the final training session.

### Randomisation and blinding

Participants are randomly assigned in a 1:1 ratio to either active or sham stimulation, using a computer-generated randomisation plan with permuted blocks of varying, undisclosed sizes to ensure allocation concealment. An independent researcher, not involved in any aspect of the trial, links the randomisation codes to the corresponding tDCS conditions and implements the allocation procedure. Consequently, both researchers and participants remain blinded to the stimulation condition. Neuropsychological test assessors, who hold at least a propaedeutic psychology degree and are trained in assessment, are also blinded and unaware of the study’s design and purpose. To evaluate blinding, assessors indicate after every assessment their perception of the study’s aim and the participant’s allocation (“Experimental,” “Control,” “No control or experimental condition,” or “I do not know”). After 16 sessions and upon intervention completion, participants and therapists report whether they believe active or sham stimulation was received. The actual treatment allocation will be disclosed only after the last participant completes the T3 assessment. During the trial, unblinding is permitted only if necessary to protect the participant’s health and safety.

### Interventions

CR and tDCS are delivered individually, twice weekly for 16–20 weeks, equating to 32–40 sessions. Given that missed sessions may occur over this period, intervention completion is defined as attending at least 20 sessions, in line with the developers’ recommendations [44]. Each session lasts 30–45 min, depending on the participant’s attention span, with tDCS applied concomitantly during the first 20 min of CR. Therapists determine the total number of sessions required based on individual progress and needs. Missed sessions are not rescheduled. Before, throughout, and after the study, participants continue to receive treatment as usual, including therapies and activities such as cognitive behavioural therapy, creative therapy, or psychomotor therapy tailored to each individual’s needs.

#### Cognitive remediation

The CIRCuiTS programme [44] employs a village metaphor and targets cognitive practice, strategy use, and metacognitive engagement. Tasks focus on attention, visual and verbal memory, and planning, and progress from abstract exercises to more complex, ecologically valid tasks, such as writing text messages or planning bus journeys. The programme and therapists suggest problem-solving strategies, track, evaluate, and discuss progress with participants. Therapists encourage monitoring and regulation of cognitive performance to foster metacognitive engagement and transfer of learned skills to everyday life. CIRCuiTS facilitates metacognitive awareness, encompassing (1) metacognitive knowledge, understanding one’s cognitive strengths and weaknesses, recognising optimal performance conditions, and implementing strategies for improved memory retention, and (2) metacognitive regulation, evaluating and adjusting this knowledge [33]. The therapy is personalised to align with the participant’s goals and performance.

Therapists, such as psychologists, rehabilitation coaches, and others with related expertise, must complete the official CIRCuiTS training programme (https://www.circuitstherapyinfo.com/training) before administering it. They receive weekly supervision from an experienced CIRCuiTS therapist during the first three months, gradually decreasing as proficiency in delivering the treatment develops, alongside monthly supervision from a clinical psychologist. This supervision ensures that therapists support participants appropriately and adhere closely to the intervention protocol.

#### Transcranial direct current stimulation

TDCS is administered using an Eldith DC stimulator (NeuroConn) targeting the DLPFC. The anode is positioned at Fp1 and the cathode at C3, following the 10–20 system, based on computational modelling with SimNIBS software [67]. Simulation results are presented in the supplementary material. Rubber electrodes (5 × 5 cm, 25 cm2) are secured with conductive paste (Ten20^®^, Weaver and Company, Aurora, CO, USA), maintaining impedance below 50 kΩ. Active stimulation consists of a 2 mA current for 1200 s, including a 30-second fade-in/out, concurrent with the first 20 min of cognitive training. This duration follows evidence suggesting that prolonged stimulation may not enhance effects [68]. In the sham procedure, the fade-in is followed by a 30-second fade-out with standard control pulses that have no therapeutic effect, to monitor electrical conductivity.

### Assessments

The researcher (NB) administers all baseline measures and sets goals with participants, test assessors administer the primary and secondary outcome measures, and a member of the research team conducts the in-depth interview.

#### Baseline measures

##### Demographic and clinical characteristics

 Baseline data include year of birth, gender, education level, primary and comorbid diagnoses, medication use, alcohol and drug use, age at first hospitalisation, and other relevant medical events or disorders.

##### Severity of clinical symptoms

 Positive, negative, and general psychiatric symptoms are assessed using the Positive and Negative Symptom Scale – brief (PANSS-6), a validated short version of the PANSS [69–72]. The PANSS-6 is developed to provide a typological and dimensional characterisation of schizophrenia, comprising six items scored on a 7-point scale, with higher scores indicating greater symptom severity. The scale includes three positive symptoms items (delusions, conceptual disorganisation, and hallucinations) and three negative symptoms items (blunted affect, social withdrawal, lack of spontaneity and flow of conversation).

##### Treatment expectancy

 Participants rate their expectations of the intervention’s effectiveness on a 1–10 scale, with 1 indicating no expected improvement and 10 indicating strong expected improvement.

#### Primary outcome measures

##### Goal attainment

 The Goal Attainment Scale (GAS) is a structured measure designed to capture self-reported recovery goals [73] and has been widely applied in research and rehabilitation practices [74–77]. Unlike trials relying solely on global outcomes, the GAS allows for a more pragmatic assessment of CR by evaluating the impact and effectiveness on individualised, real-world goals. Participants define up to a maximum of three individual recovery goals during an interview. Each goal is scored between − 2 and 2, with higher scores indicating better performance, and weighted according to importance and difficulty. Evaluations occur at subsequent assessments using a GAS-weighted T-score derived from recent research methods [78] and approaches used in a large CIRCuiTS trial [77].

##### Daily living skills

 The Independent Living Skills Survey (ILSS) measures the basic functional living skills and has acceptable psychometric characteristics [79, 80]. Both the Self-Report (ILSS-SR) and the Informant (ILSS-I, completed by the participants’ case manager) versions are utilised. The ILSS-SR comprises 70 items, nine of which are interviewer-rated, scored as yes, no, or not applicable. The ILSS-I includes 102 items rated from 0 (never) to 4 (always). Total scores are summed and averaged per domain, with higher scores indicating better performance.

#### Secondary outcome measures

Cognitive outcomes are assessed across multiple domains, with at least one test per MATRICS domain, except for social cognition, which is not targeted in this trial.

##### Processing speed

The Controlled Oral Word Association Test (COWAT) assesses verbal fluency and processing speed [81]. Participants must generate as many words as possible beginning with a specified letter within 60 s. Performance is the total number of words across three trials, and higher scores reflect better performance.

##### Attention, processing speed, and mental flexibility

The Trail Making Test (TMT) assesses visual search abilities, processing speed, and mental flexibility [82]. In version A, participants connect digits sequentially; in version B, digits and letters are alternated. Version B is relatively more challenging, as it also tests mental flexibility. Completion times are recorded, with longer times indicating lower performance.

##### Attention and working memory

The Digit Span forward task assesses immediate auditory attention [83]. The test administrator reads sequences of digits aloud, gradually increasing the length of each sequence, while participants repeat the digits in the same order. The score reflects the number of correctly recalled sequences, with a maximum score of 16 points. The Digit Span backward task assesses working memory, requiring participants to repeat the digits in reverse order, with a maximum score of 14 points [83]. In both versions, higher scores correspond to higher performance.

##### Visual memory

 The Visual Reproduction subtest of the Wechsler Memory Scale – Fourth edition (WMS-IV) assesses visual memory for abstract designs [84]. Participants are presented with five designs for 10 s each and reproduce them from memory. Each design is evaluated on different criteria, with a maximum score of 43 achievable. A recognition task involving the same designs is conducted after 30 min, with a maximum score of 5. Higher scores correspond with higher performance.

##### Verbal memory

 The 15-word learning task (WT-15) measures verbal memory [85, 86]. The test administrator reads a list of 15 words, and participants are asked to recall as many words as possible. This procedure is repeated five times, and the total number of correctly reproduced words constitutes the score. After 15–20 min, the delayed recall is administered, with a maximum score of 15. Higher values indicate higher performance on both immediate and delayed recall.

##### Executive functioning

 Subtasks of the Behavioural Assessment of the Dysexecutive Syndrome (BADS) assess executive functioning. First, the Key Search task assesses planning and organisation [87]. Participants are asked to plan a search route for an imaginary lost key within a field. Scores reflect whether the search strategy is systematic, efficient, and effective, and they receive deductions for slow responses. Second, the Dysexecutive questionnaire (DEX), a 20-item measure, evaluates metacognitive performance on executive functioning skills [87]. Both versions, filled in by participants and their case managers, are administered. Each question is scored between 0 (never) and 4 (very often). Higher total scores indicate worse performance on both subtasks.

##### Reasoning and problem-solving

 The Stroop Colour and Word Test (SCWT) assesses processing speed, cognitive flexibility, and cognitive control [88]. Participants first read colour blocks and colour names aloud (two baseline tasks). They then name the ink colour of incongruent colour words (interference), requiring suppression of the habitual response to facilitate an unusual one. More time needed corresponds to lower performance. The Stroop interference effect is calculated as the increase in completion time from the baseline to the interference task, providing a general measure of executive functioning. The interference score is calculated by the equation: (colour-word reaction time) – ((colour reaction time + word reaction time)/2).

##### Subjective cognitive functioning

 The Cognitive Failure Questionnaire (CFQ) measures subjective cognitive functioning [89, 90] and includes 25 questions assessing attention and memory. Each question is scored between 0 (never) and 4 (very often), with higher total scores indicating more cognitive mistakes.

##### Observed cognitive functioning

 The Nurses’ Observation Scale of Cognitive Abilities (NOSCA) is a behavioural rating scale to examine cognitive abilities in service users, completed by participants’ case managers [91]. It consists of eight subscales, representing subdomains of cognition, using 39 items scored between 0 and 3, with higher total scores indicating better functioning.

##### Negative symptoms

 The Self Evaluation of Negative Symptoms (SNS) is a 20-item questionnaire assessing negative symptoms from the perspective of the service user [92] categorised in the five domains of negative symptoms – social withdrawal, diminished emotional range, avolition, anhedonia, and alogia. Items are scored on a Likert scale ranging from 0 (strongly disagree) to 2 (strongly agree), with higher scores indicating greater severity.

##### Self-efficacy

The Dutch version of the General Self-Efficacy Scale (GSES) comprises 10 items that evaluate self-efficacy, or the respondent’s general perception of their ability to achieve goals, overcome challenges, and perform well on tasks [93]. Participants rate items on a 4-point response scale from 1 (strongly disagree) to 4 (strongly agree). Responses are averaged to produce an overall score, and higher scores indicate greater self-efficacy.

##### Social functioning

The Social Functioning Scale (SFS) is a 79-item standard measure developed and validated for individuals with schizophrenia, demonstrating appropriate psychometric properties [94]. It includes subscales assessing social engagement or withdrawal, interpersonal functioning, current social activities, recreational activities, independence-competence, independence-performance, and employment. Both the observer-rated and self-rated versions are used, with higher scores indicating higher social functioning. Additionally, the Behapp smartphone application (www.behapp.com) objectively and passively measures social functioning in daily life, capturing sociability and social exploration [95]. After installation and activation with a unique code, Behapp runs in the background of participants’ smartphones, collecting data on communication events (e.g., incoming and outgoing calls), application usage (e.g., social or entertainment applications), and GPS-based location. Data are gathered in four 14-day phases aligned with T0-T3, and gathering is stopped automatically after completion of each phase. All information is encrypted, stored locally until secure upload, and the content of calls, messages, or applications is never recorded. Participants provide explicit consent for Behapp during inclusion, and opting out of Behapp does not affect study participation. Behapp has already proven feasible for use in individuals with schizophrenia [96].

#### In-depth interview

To examine service users’ metacognitive skills and subjective experiences, we use an adapted version of the Indiana Psychiatric Illness Interview (IPII), a semi-structured interview designed originally to elicit illness experiences and spontaneous metacognitive thoughts, which lasts 30–60 min [97]. For this study, the interview is revised to focus on cognitive experiences and everyday cognitive functioning. Responses are rated with the abbreviated Metacognitive Assessment Scale (MAS-A) as a rating scale [98, 99], adapted for this study to assess the metacognitive ability of cognitive functioning. The main adaptations in the IPII and MAS-A involved refocusing the content from mental illness to cognitive abilities, simplifying the language, and dividing complex items into shorter segments to improve accessibility. The MAS-A evaluates self-reflectivity, understanding the other’s mind, decentration, and mastery, and has consistently shown robust validity and reliability [98]. Higher scores on each subscale indicate a greater ability to form complex representations of oneself and others.

Additional open-ended questions explore subjective experiences with CIRCuiTS, its perceived impact on metacognition, daily functioning, supportive components, and potential areas for improvement.

A focus group with CIRCuiTS therapists further evaluates the in-depth interview. Based on established guidelines for focus group discussion research [100], this discussion may lead to further refinement of the interview scheme.

### Adverse event reporting

TDCS is regarded as a safe method with no evidence of short- or long-term harm when applied within established parameters [101]. Accordingly, the risk of adverse events is expected to be low. During each session, therapists will systematically document any participant-reported complaints and potential tDCS-related side effects (e.g., burning, headache, itching, tingling). All adverse events reported by participants, researchers, or health professionals will be assessed for seriousness and reported to the relevant parties in accordance with the Medical Ethics Review Committee of the University Medical Centre Groningen (METc) procedure.

### Participant timeline

An overview of enrollment, intervention, and assessments is shown in Table 1.

**Table 1:** Schedule of enrolment, interventions, and assessments

Timepoint	Enrolment	Pre-intervention		Intervention	Post-intervention	6-months follow-up
	< T_0_	T_0_	T_1_		T_2_	T_3_
Enrolment						
Eligibility screen	x					
Informed consent	x					
tDCS safety checklist	x					
Randomisation						
Allocation			x			
Interventions						
CR + active tDCS				x		
CR + sham tDCS				x		
Assessments						
PANSS-6		x				
GAS		x (goals set)	x (outcome rated)		x (outcome rated)	x (outcome rated)
ILSS-I & -SR		x	x		x	x
COWAT		x	x		x	x
TMT-A & -B		x	x		x	x
BADS key search		x	x		x	x
WMS-IV visual reproduction		x	x		x	x
Digit Span (forward/backward)		x	x		x	x
WT-15		x	x		x	x
SCWT		x	x		x	x
CFQ		x	x		x	x
DEX-S & -I		x	x		x	x
NOSCA		x	x		x	x
SNS		x	x		x	x
GSES		x	x		x	x
SFS		x	x		x	x
Behapp		x	x		x	x
In-depth interview					x	

*BADS* Behavioural Assessment of the Dysexecutive Syndrome, *CFQ* Cognitive Failure Questionnaire, *COWAT* Controlled Oral Word Association Test, *CR* cognitive remediation, *DEX−S & −I* dysexecutive questionnaire – self & informant, *GAS* Goal Attainment Scale, *GSES* General Self−Efficacy Scale, *ILSS−I & −SR* independent living skills survey – informant & self−report, *NOSCA* nurses’ observational scale of cognitive abilities, *PANSS−6* The Positive and Negative Syndrome Scale – brief, *SCWT* Stroop Colour and Word Test, *SFS* Social Functioning Scale, *SNS* self−evaluation of negative symptoms, *tDCS* transcranial direct current stimulation, *TMT* Trail Making Test, *WMS−IV* Wechsler Memory Scale – Fourth edition, *WT−15* 15−word learning task

### Proposed sample size and power calculation

The sample size was determined for the primary objective, namely the within-subject effect of CR on daily functioning compared with the waitlist period in the non-concurrent multiple baseline design, pooled across tDCS conditions. The calculation was based on an anticipated effect size of d = 0.36 for daily functioning, derived from a prior study in the same target population using a compensation-focused CR approach [102]. Using a paired t-test for dependent groups (two-sided α = 0.05, power = 0.80, Cohen’s d = 0.36), a sample size of 50 participants per group is required. To account for an estimated dropout rate of 20%, we aim to recruit a total of 126 participants, equating to 63 individuals per group. Detailed descriptive outcome data from our pilot study are available in the Open Science Framework (https://osf.io/cj49e/) [65]. Pilot-derived effect-size estimates for everyday functioning were not used for formal powering because functional outcome assessment in the pilot was heterogeneous, and did not capture participant-reported benefits, suggesting insufficient sensitivity to detect CR-related change in this setting. Therefore, we based the effect size on prior evidence from the larger study mentioned above [102], which provides a more precise estimate.

The sample size for the in-depth interviews is determined by the participants’ willingness to participate and thematic saturation. Ideally, the sample size ranges from 10 to 20 participants, given the homogeneous nature of the sample and the narrow scope of the study topic. Data collection ceases once data saturation is achieved.

### Statistical analysis

Data analyses will follow an intention-to-treat approach using multilevel models implemented in R (‘lme4’ or ‘glmer’ packages). This approach accounts for the nested structure of repeated measures within participants and participants nested within centres. Model assumptions will be assessed using scatter plots of residuals and Quantile-Quantile (QQ) plots. Multilevel models are generally robust to moderate violations of normality. Models will be estimated using maximum likelihood, which uses all available data under a missing-at-random assumption.

We will build separate multilevel models for each primary and secondary outcome. Timepoint will be included as a categorical within-subject variable with four levels (T0 = baseline; T1 = pre-intervention; T2 = post-intervention; T3 = follow-up). Centre and tDCS condition (active vs. sham) will be included as between-subject variables. Given the staged recruitment, cohort will be included as a fixed-effect covariate to account for cohort-related differences over time (e.g., staff changes or evolving site practices). Models will include a random intercept for baseline differences across individuals, and, where supported, a random slope for timepoint will allow individual trajectories over time (simplified if convergence issues arise). Fixed effects will be estimated for timepoint, tDCS condition, centre, cohort, and their relevant interactions. We will set T1 (pre-intervention) as the reference level to be able to compare T0 vs. T1 (changes during waitlist period), T2 vs. T1 (changes during intervention), and T3 vs. T1 (longer-term changes).

The primary CR effect will be evaluated by comparing changes observed during the intervention period with changes during the waitlist period, pooling across tDCS conditions. The tDCS augmentation hypothesis will be tested via the intervention × timepoint interaction, operationalised as the difference in change from T1 to T2 and from T1 to T3 between active tDCS and sham tDCS. Given the trial is powered for the primary within-subject CR effect rather than the between-group tDCS contrast, tDCS results will be interpreted as secondary and hypothesis-generating, emphasising effect size estimates and 95% confidence intervals.

We have three primary outcomes (GAS, ILSS-O, ILSS-SR). To control multiplicity, we will control the family-wise Type I error rate across the three primary outcomes at two-sided α = 0.05 using the Holm correction.

#### Data from Behapp

The raw data will be pre-processed by the Behapp team, producing a pseudo-randomised report of behavioural outcomes related to participants’ communication and mobility. Separate multilevel models, as described above, will be built for each outcome (e.g., unique places visited, times opening communication apps, number of incoming or outgoing calls) to assess the effects of CR during the waiting period and to determine whether CR + active tDCS enhances these effects.

#### Thematic analysis

Data from the in-depth interview will be transcribed and analysed using thematic analysis [103, 104], following established guidelines for considering the perspectives of service users. Both deductive and inductive approaches will be applied: an initial codebook will be developed based on the literature, while new codes and themes will emerge from the data. The initial codebook will cover themes such as goals, metacognitive knowledge and regulation, daily functioning, and confidence. Results will be presented as a coherent narrative, illustrated with direct quotes from participants.

### Research ethics

This research project was approved by the METc (2023/434) in accordance with the latest revision of the Declaration of Helsinki [105] and was pre-registered in ClinicalTrials.gov (NCT06378463).

### Data management

Data will be stored digitally on a secure University of Groningen drive for 15 years. Files containing personal identifiers will be stored separately with restricted access. Data will be entered into electronic case report forms that mirror the paper forms and will undergo independent verification checks by trained staff. Data quality will be supported through consistency checks, and changes to the database will be logged. Detailed data handling procedures are described in the study Data Management Plan (DMP) stored at the University of Groningen.

### Confidentiality

Only personal information required for screening, informed consent, and scheduling will be collected. Participants will be identified using unique study IDs used on all study documents concerning that participant. Apart from the signed consent form and proof of payment, no study materials will contain directly identifying information. The link between identifiers and study IDs will be maintained in a password-protected key file stored in a secure, restricted-access location, and access to this key file will be limited to researchers directly involved in data collection. Other study staff will be provided only with pseudonymised or fully anonymised datasets that cannot be traced back to an individual, and only anonymised data will be used for publications. Confidentiality will be protected before, during, and after the trial through restricted access in compliance with the Dutch General Data Protection Regulation (GDPR).

### Monitoring

#### Data monitoring committee

A data monitoring committee will not be appointed because the interventions are considered safe.

#### Trial monitoring

The researchers of this study coordinate and steer the trial. LM is the principal investigator. The project management group (NB, AP, and LM) will provide day-to-day support. NB is responsible for identifying potential participants, recruitment, inclusion procedures (e.g., informed consent), data collection, and maintaining participant retention during the waitlist period. AP and LM will supervise NB and monitor study progress and conduct. AP and GM will supervise the delivery of the CR training, and AP and BC will supervise the delivery of tDCS. The progress of the study will be discussed on a monthly basis with the whole research team. All authors will contribute to writing the manuscript.

#### Service user involvement

A user committee comprising service users, relatives, caregivers with clinical practice experience, educators, and managers is established to advise the research team throughout the trial. The committee provides guidance on trial design, recruitment strategies, and the interpretation and dissemination of results, while offering insights from client and clinical perspectives to enhance client-centredness and trial effectiveness. The research team organises online meetings at least annually, with additional sessions scheduled as needed.

#### Protocol amendments

Substantial protocol amendments affecting study conduct, participant benefit, or safety will be reported to participants, the trial registry, the ethics committee, and the sponsor, whereas minor clarifications will be logged in an amendments list.

### Dissemination

The results of this trial will be publicly disclosed. Results will be presented at (inter-)national conferences and involved study sites. Results will be published in scientific and non-academic journals to reach researchers, clinicians, clients, and their relatives.

## Discussion

The HEADDSET+ study is a pragmatic, randomised, sham-controlled, multi-centre trial evaluating the effectiveness of CR alone and in combination with tDCS in individuals with SMI living in sheltered housing. By targeting goal attainment, daily functioning and cognitive functioning, this study aims to improve independence, recovery, and overall quality of life. CR provides structured cognitive training that supports functional improvements, while tDCS may increase neuroplasticity. TDCS may modestly augment the effects of CR, although current evidence is mixed, and effects appear small [62, 64]. If proven effective, this combined intervention can be integrated into standard care, providing an effective treatment for individuals with SMI requiring long-term support.

This trial builds directly on our earlier pilot study [66] by expanding the sample size, modifying inclusion criteria, and optimising outcomes. The primary outcome, the Goal Attainment Scale (GAS), offers a standardised method to capture progress on personalised goals, typically formulated at the level of cognitive functioning, daily functioning, or participation, thereby ensuring functional relevance and promoting adherence [73]. Our pilot trial showed that participants experienced difficulties setting goals, underscoring the importance of using a structured instrument, such as the GAS, to form goals prior to starting CR, consistent with earlier research [75, 76, 106]. Secondary measures include questionnaires on metacognition and self-efficacy to clarify potential treatment effects, alongside self- and observer-rated questionnaires to provide insight into cross-perspective agreements. Finally, the Behapp smartphone application adds an innovative, objective complement to traditional self-report measures through real-time monitoring of social and daily activities.

This is among the first large-scale clinical trials to systematically evaluate CR combined with tDCS, moving beyond prior small pilot studies [65]. Several design features strengthen its scientific and practical value. First, the multi-centre approach increases the generalisability of the results. Second, regular supervision by an experienced clinical therapist will provide fidelity and consistency in intervention delivery. Last, the within-subject design allows participants to serve as their own controls through the waiting period. While the absence of a separate control group receiving only CR or an active control condition is a limitation, this design is particularly advantageous due to the heterogeneity of the target population, the higher feasibility because fewer participants are needed, and the focus on individualised goals.

Clinical trials in this population often face challenges such as recruitment and retention difficulties, particularly among individuals with low motivation or residing in less supportive environments. Insights from our pilot study enabled proactive strategies to anticipate and address these challenges. Despite concerns about recruitment, retention, or discomfort with tDCS posing barriers, particularly in a population with symptoms such as lack of trust or suspiciousness, the pilot demonstrated high acceptability and minimal attrition. In the present trial, active involvement of service users ensures alignment with the needs and experiences of the target population, which may improve both trial design and recruitment strategies. To illustrate, participants receive detailed information about tDCS and its potential side effects, and trainers monitor well-being throughout the sessions. If a participant refuses stimulation during a session, CR continues without tDCS, with reattempts in later sessions. Regular contact during the waiting period supports engagement, and offering home-based sessions when needed provides flexibility to maintain it further. Additional challenges include using research staff as trainers rather than professionals embedded within routine care. While this ensures consistent delivery of CR, it may limit direct transfer to clinical practice. To mitigate this, healthcare professionals are actively involved, and strategies are pursued to stimulate integrations of CR into standard practice.

This trial addresses a significant gap in the literature, as few trials have explored the effects of CR in individuals with SMI in sheltered housing. Also, the effects of tDCS on functional outcomes or its integration with CR programmes encompassing all four core elements remain limited [23, 64]. By systematically evaluating this combination in a large clinical sample, HEADDSET+ will determine whether CR improves real-world outcomes in a group with substantial unmet clinical needs and whether adjunctive tDCS enhances these effects. Ultimately, findings may lead to broader integration of CR and tDCS into clinical and rehabilitation practice, ensuring that individuals with SMI have access to effective, evidence-based interventions promoting recovery.

### Trial status

Recruitment is ongoing. Participant enrolment commenced in May 2024, with an estimated study completion date of May 31, 2027. At the time of submission, we have enrolled 59 participants at three study sites.

## Supplementary Information

Below is the link to the electronic supplementary material.

Supplementary Material 1

## Data Availability

No datasets were generated or analysed during the current study.
