# Supplementary material for: Improving functioning in severe mental illness with cognitive remediation: study protocol for a pragmatic randomised multi-centre trial with adjunct sham-controlled tDCS (HEADDSET+)
Source: Eur Arch Psychiatry Clin Neurosci. 2026 Apr 24;276(5):2085–99. doi: 10.1007/s00406-026-02245-7 (PMC13481488; doi:10.1007/s00406-026-02245-7)
Supplement: Supplementary file 1 [file 406_2026_2245_MOESM1_ESM.docx]

**Supplementary material**

**Improving Functioning in Severe Mental Illness with Cognitive Remediation: Study Protocol for a Pragmatic Randomised Multi-Centre Trial with Adjunct Sham-Controlled tDCS (HEADDSET+)**

Nienke Catharina Buist^1,2,3^, Anika Poppe^4^, Branislava Ćurčić-Blake^3,5^, Gerdina Hendrika Maria Pijnenborg^1,3,6,7^, Lisette van der Meer (corresponding author, l.van.der.meer@rug.nl)^1,2,3,7^

Affliations:
 ^1^Department of Clinical and Developmental Neuropsychology, University of Groningen, Grote Kruisstraat 2/1, 9712 TS Groningen, The Netherlands. ^2^Department of Rehabilitation, Lentis Psychiatric Institute, Lagerhout E35, 9741 KE Zuidlaren, The Netherlands. ^3^Research School of Behavioural and Cognitive Neurosciences (BCN), University of Groningen, Groningen, The Netherlands. ^4^Department of Psychiatry, Donders Institute for Brain, Cognition and Behaviour, Radboud University Medical Center, The Netherlands. ^5^Department of BSCS Neuroscience, University of Groningen, University Medical Center Groningen, Groningen, The Netherlands. ^6^Department of Psychotic Disorders, GGZ Drenthe, Assen, The Netherlands. ^7^Rob Giel Research Center, University Center Psychiatry, University Medical Center, University of Groningen, Groningen, the Netherlands.

**Current Flow Modelling**

Electrode locations were informed by computational modelling in SimNIBS ^[1]^, which estimates the distribution of current flow and the resulting electric field for a given tDCS montage using realistic head models derived from structural MRI data. Following the same modelling approach as in our pilot study, we compared several commonly used montages to identify those most likely to target the left dorsolateral prefrontal cortex (DLPFC). The left DLPFC was selected a priori because it is a key area within fronto-parietal networks that support cognitive task performance ^[2]^ and are frequently affected in individuals with schizophrenia-spectrum disorders ^[3]^.

For the simulations, we used structural MRI scans from five participants from our earlier pilot study conducted in the same target population. To capture variability in head and tissue characteristics, these participants were selected to represent a range of body mass index (BMI): one underweight, two average weight, and two overweight. BMI was used as a proxy for subcutaneous fat level, a physiological factor that can influence current flow ^[4]^. Table S1 summarises the mean electric field estimated in the left DLPFC across montages. The highest mean electric field was observed for the montage with electrodes at Fp1 and C3 (2 mA). Figure S1 illustrates the corresponding electric field distribution for one representative participant.

| **Table S1.** Mean electric fields in the left DLPFC (-38, 44, 26) in V/m | | | | | |  |
| --- | --- | --- | --- | --- | --- | --- |
| Subjects | C3-Fp1 | C3-Fp2 | C5-Fp1 | C5-Fp2 | F3-F4 | F3-Fp2 |
| S1 | 0.4306 | 0.3780 | 0.3871 | 0.3700 | 0.2261 | 0.2355 |
| S2 | 0.4093 | 0.3528 | 0.3653 | 0.3392 | 0.1695 | 0.1769 |
| S3 | 0.3866 | 0.3498 | 0.3404 | 0.3397 | 0.2129 | 0.2210 |
| S4 | 0.3444 | 0.3125 | 0.3036 | 0.3058 | 0.2098 | 0.2051 |
| S5 | 0.3434 | 0.3152 | 0.3054 | 0.3050 | 0.1880 | 0.1999 |

**Figure S1.** Illustration of electric field distribution (magE in Volt per meter, V/m; electrode_currents in Ampère, A)

**References**

1. Saturnino, G. B., Puonti, O., Nielsen, J. D., Antonenko, D., Madsen, K. H., & Thielscher, A. (2019). *Brain and Human Body Modeling, Computational Human Modeling at EMBC 2018*. 3–25. <https://doi.org/10.1007/978-3-030-21293-3_1>

2. Cole, M. W., Reynolds, J. R., Power, J. D., Repovs, G., Anticevic, A., & Braver, T. S. (2013). Multi-task connectivity reveals flexible hubs for adaptive task control. *Nature Neuroscience*, *16*, 1348–1355. <https://doi.org/10.1038/nn.3470>

3. Sheffield, J. M., Repovs, G., Harms, M. P., Carter, C. S., Gold, J. M., MacDonald, A. W., Ragland, J. D., Silverstein, S. M., Godwin, D., & Barch, D. M. (2015). Fronto-parietal and cingulo-opercular network integrity and cognition in health and schizophrenia. *Neuropsychologia*, *73*, 82–93. <https://doi.org/10.1016/j.neuropsychologia.2015.05.006>

4. Li, L. M., Uehara, K., & Hanakawa, T. (2015). The contribution of interindividual factors to variability of response in transcranial direct current stimulation studies. *Frontiers in Cellular Neuroscience*, *9*, 181. <https://doi.org/10.3389/fncel.2015.00181>
